# Employing antineutrino detectors to safeguard future nuclear reactors from diversions

**DOI:** 10.1038/s41467-019-11434-z

**Published:** 2019-08-06

**Authors:** Christopher Stewart, Abdalla Abou-Jaoude, Anna Erickson

**Affiliations:** 1 Nuclear and Radiological Engineering Program, Georgia Institute of Technology, Atlanta, GA 30332 USA; 2 University of California, Berkeley, CA 94720 USA; 3 Idaho National Laboratory, Idaho Falls, ID 83402 USA

**Keywords:** Nuclear fusion and fission, Energy policy

## Abstract

The Non-Proliferation Treaty and other non-proliferation agreements are in place worldwide to ensure that nuclear material and facilities are used only for peaceful purposes. Antineutrino detectors, sensitive to reactor power and fuel changes, can complement the tools already at the disposal of international agencies to safeguard nuclear facilities and to verify the States’ compliance with the agreements. Recent advancement in these detectors has made it possible to leverage them to reduce the likelihood of an undetected diversion of irradiated nuclear material. Here we show the sensitivity of antineutrino monitors to fuel divergence from two reactor types: a traditional light-water reactor and an advanced sodium-cooled reactor design, a likely candidate for future deployment. The analysis demonstrates that a variety of potential diversion scenarios can be detected by such a system. We outline recent developments in monitoring capabilities and discuss their potential security implications to the international community.

## Abstract (web summary)

Nuclear reactors can be used for energy generation or for dangerous weapons and therefore their monitoring is crucial. Here the authors discuss detecting antineutrino from a nuclear reactor and use it for nuclear safeguards in a diversion scenario.

## Introduction

Nuclear energy is touted as a key tool for providing high-volume, low-carbon energy^1,2^, with about 450 nuclear power plants currently producing around 11% of world’s energy and 61 additional plants under construction^3^. As the number of nuclear energy-producing nations grows and the nuclear fuel cycle becomes ever more complex and internationalized, stresses on the existing safeguards infrastructure will only increase. With new, unconventional types of reactors introduced to the market, standard approaches to nuclear proliferation safeguards also become less reliable.

A strongly advocated means of containing proliferation is to establish a provider-user fuel cycle, in which front-end and back-end processes are made available by states with such infrastructure already in place. These capabilities, specifically fuel enrichment and reprocessing, provide a direct path for a state to develop a nuclear weapon. A provider-user approach removes the incentive for user states to develop these proliferation-sensitive technologies. Existing agreements (for example, between the U.S. and the United Arab Emirates)^4^ accomplish this by legally binding nations not to pursue enrichment or reprocessing technology. More novel approaches rely on a hub-spoke model^5,6^: advanced reactors of battery-type, which rely a single but long (10–30 years) fuel loading and usually have relatively low power, are leased to the user state and returned to the provider state at the end of their lifetime for disposal or recycling of spent fuel. While these proposals reduce proliferation risks associated with reactor fuel cycle, they do not eliminate it. Plutonium produced as a by-product of normal operation in any reactor could still be diverted for use in weapons^7^.

Traditional safeguards and inspections have worked well, deterring material diversion from current reactors for many decades. The International Atomic Energy Agency (IAEA) relies on inspector presence during reactor refueling intervals, as well as seals on fuel assemblies to keep track of weapon-usable material. However, these checks are not infallible, since inspectors are not always present on a site and can be refused entry. Future reactor developments on the horizon are likely to strain the nonproliferation regime further. The shift in the industry towards more distributed generation using Small Modular Reactors (SMR) will stretch out monitoring resources. Advanced so-called “Generation IV” reactor designs often do not require any refueling or, conversely, continuously refuel their cores, complicating inspections efforts and requiring continuous monitoring of the fuel state. The development of long-life reactors also raises additional questions about safeguards, notably regarding when and how inspections should be conducted. Inspectors cannot rely on refueling cycles to monitor these reactors as they typically do for Pressurized Water Reactors (PWR), which operate on an 18–24-month cycle. The issue of inspections is exacerbated by the weapon-grade quality of plutonium in such cores (<7% ^240^Pu) resulting from the fast neutron spectrum that is necessary to breed fissile material in the core and subsequently burn it in situ without loss of reactor criticality. Future safeguarding technologies deployed to combat these challenges will ideally possess always-on, real-time monitoring attributes and ensure the integrity of the collected data against attempts to falsify it.

**Fig. 1:**
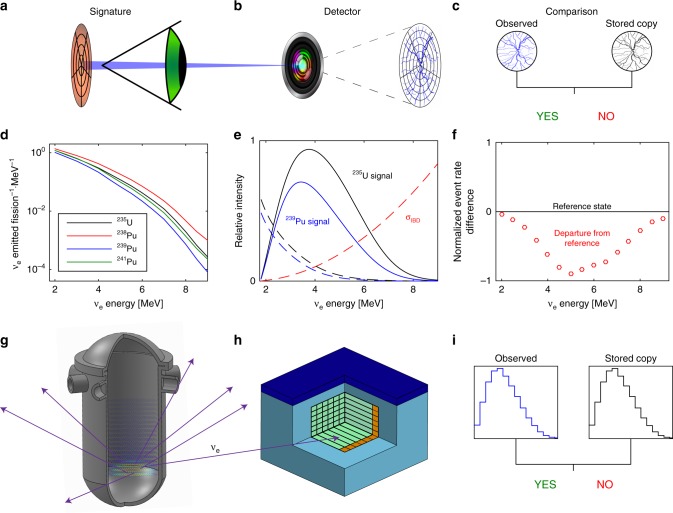
Principle of reactor operation verification with antineutrino monitors. The process for verifying reactor inventory integrity with antineutrinos bears similarities to biometric scans such as retinal identity verification. In retinal scans, an infrared beam traverses a person’s retina (**a**) and the blood vessels, distinguishable by their higher light absorption (**b**) relative to other tissue, are mapped. The mapping is extracted and compared to a copy stored in a database (**c**), and if the two match, the person’s identity is verified. Similarly, a nuclear reactor (**g**) continuously emits antineutrinos which vary in flux and spectrum with the particular fuel isotopes undergoing fission (**d**). Some interact in a nearby detector (**h**) via inverse beta decay (**e**). The measured signal is compared to a reference copy stored in a database for the relevant reactor, initial fuel, and burnup (**f**); a sufficiently matching signal indicates that the core inventory has not been covertly altered. If the antineutrino flux of a perturbed reactor is sufficiently different from expected, a diversion can be concluded to have taken place (**i**)

One potential route to meeting such continuous safeguarding requirements can be provided by antineutrino particles, a by-product of the nuclear fission process. Indeed, advances in antineutrino detector capabilities can yield a continuous, unobtrusive, and unfalsifiable way of obtaining information on a nuclear core. In this paper, such a system is termed Reactor Evaluation Through Inspection of Near-field Antineutrinos, or RETINA (see Fig. 1).

At a 2008 workshop by the IAEA’s Division of Technical Support, experts evaluated how antineutrino detectors can improve the monitoring capabilities of the organization and complement the role of inspectors^8^. The report concluded that RETINA systems could complement safeguard capabilities by providing an independent, real-time measure of the reactor power and of its fissile inventory. Many antineutrino systems have been built and operated in the past to demonstrate some of these safeguarding capabilities, notably at SONGS^9^, Daya-Bay^10^, and Nucifer^11^. The SONGS project in particular, has demonstrated how near-field monitoring of reactors can deduce information about operational status, by leveraging the proportionality of antineutrino flux to the power output of a reactor^12^. The results showed that operational status (on/off) can be detected with a greater than 99% confidence within just 5 h. Power fluctuations were measured on month-long scales with an estimated 8.3% precision. Obtaining information on evolution of the core content has been demonstrated using computer simulations, as well as in antineutrino physics experiments showing how modifications to the isotopic composition result in noticeable changes in antineutrino spectral yields. For example, a study on the North Korean 1994 crisis showed that diversions of 8-kg-worth of plutonium could be detected within 90 days at a 90% confidence level^13^. Another study on a mixed-oxide core (MOX) showed that modifications to the isotopic composition (including between different levels of plutonium purity) can be detected using a RETINA-based system with a high degree of confidence within a single fuel cycle (18 months)^14^.

In the following, we examine the performance of antineutrino detectors when employed as diversion monitors of reactors with distinct operational characteristics. Antineutrinos interact with matter with such rarity that it would take nearly a light-year’s worth of lead to shield a detector from the antineutrinos emitted by a nuclear reactor. While the probability of interaction remains small, an operating reactor generates ample quantities of antineutrinos from beta decay of fission products in the fuel—on the order of 10^21^ per GWh of electricity produced. As a result, a distinct antineutrino signature can be observed with a relatively small above-ground detector^9,15^. Here, we examine the efficacy of antineutrino detectors under several scenarios in which fissile material in the range of one to a few significant quantities (SQ) of plutonium has been diverted from either an established advanced pressurized water reactor (PWR) or a more exotic example of a long-life core design, the Ultralong Cycle Fast Reactor (UCFR-1000)^16^, with more details of modeling and analysis provided in Supplementary Note 2. We calculate the reference signal which an antineutrino detector monitoring of normal burnup behavior of each core should produce under nominal operating conditions. We then compare these to the estimated detector data from each of the diversion scenarios. The time required for a monitoring body to conclude that the reactor state differs from its declared configuration is evaluated against proliferation breakout timeframes as indicated by IAEA timeliness criteria. For a technically sophisticated actor, the IAEA estimates a reasonable limit of three months for the weapon-conversion time by after the occurrence of a material diversion^17^. We show that, for the path to a plutonium-based weapon, actionable information for some scenarios can be obtained prior to breakout periods, even for relatively small changes in reactor composition.

## Results

### Modeling the reactor antineutrino signature

The most mature technology for detecting electron antineutrinos relies on the reverse of the *β*^−^ reaction characteristic of fission products. In this process, aptly named inverse beta decay (IBD), an antineutrino interacts with a proton producing a positron and a neutron that are readily detectable with standard technologies. The IBD reaction has an extremely low probability (on the order of 10^−43^ cm^2^) of occurrence and requires an antineutrino to have a threshold energy of 1.8 MeV. The threshold reduces the number of detectable antineutrinos produced per fission to approximately 1.92 for ^235^U and 1.45 for ^239^Pu. More details with respect to IBD interactions can be found in Supplementary Note 1. The plot under section (b) of Fig. 1 shows the absolute distribution of emitted antineutrinos per fission event per MeV of energy. The difference in antineutrino emitted for various fissile isotopes results in a distinctive pattern of the fuel composition as a function of time. For example, the plot in section (c) of Fig. 1 shows the difference in antineutrino signatures if the reactor core is exclusively composed of ^235^U (as in case of freshly loaded reactor core with no irradiated fuel present) or ^239^Pu (reactor operating on so-called mixed-oxide fuel designed to dispose of plutonium) at the beginning of operation. These two cases are extrema and are rarely present in actual reactors; however, interpolation is possible providing the necessary information about the core composition for verification purposes.

Due to the small antineutrinos interaction probabilities, only a few counts are registered in the detector per day (of the order of 100–1000, depending on reactor power, detector size, efficiency, and placement away from the core). Therefore, reaching conclusive statistical evidence of modifications to the core operations requires timelines from the order of hours to months, depending on the information needed. A small scintillator-based detector (with an active volume on the order of 1 to 5 m^3^) positioned at 17 to 25 meters away from the reactor core (typical distances associated with the reactor containment), can notice complete shutdowns in the order of a few hours, but small modifications to the fuel composition of a core can take months to detect. Still, RETINA systems could provide a notable advantage over the current regime where inspections normally take place every 18 months and depend on tamper-evident seals (markers to identify if an object was manipulated or moved during the absence of inspectors) to establish continuity of knowledge. A monitoring body with access to RETINA information can place bounds on the material inside the reactor without requiring a cessation of operations, radiation-hardened electronics, or even the physical presence of inspectors. In fact, the ability to detect large changes in power level within hours^12^ and to observe the isotopic evolution of reactor fuel as it proceeds through its burnup cycle^12^ has already been demonstrated experimentally at the whole-core scale using an IBD-type detector at a nuclear reactor cite^18^.

The detectors selected for the analysis were based on the PROSPECT AD-I and AD-II designs^19^. The initial phase detector (AD-I) uses a 3 ton segmented ^6^Li-doped liquid scintillator detector at a distance of 7–12 m from the core. The second phase is a 10 ton detector at a distance of 15–19 m. The experiment objective is to monitor the antineutrino signature from a highly enriched uranium (HEU) fueled reactor at the Oak Ridge National Laboratory. A total of three PROSPECT-like detectors were used in each of the PWR and UCFR analyses. Each with a fiducial mass of 5 tones, placed at a 25 m radial distance from the core, and with 120° azimuthal intervals. In the case of the PWR, the detectors were all placed at the same axial location. For the UCFR, the three detectors were staggered at different heights to better account for the axial core evolution in the reactor: the first even with the bottom of the core, the second at core midplain, and the third at the top. The detector parameters are summarized in Table 1.

**Table 1:** Characteristics of antineutrino detector system

Parameter	Value
Fiducialized target mass (*t*)	5
Proton density (no./m^3^)	5.5 × 10^28^
Efficiency in fiducial volume (%)	42
Energy resolution	4.5%/$\sqrt E$
Distance to core (m)	25

The parameters are based on the PROSPECT design^19^

The energy spectrum of the detector signal was divided into 0.5 MeV bins, as finer energy discretization was observed to produce negligible gains in precision during preliminary analysis. The detector event rate in each bin, *n*_*i*_, can be calculated as:

1 $$n_i = N_{\mathrm{D}}\Sigma _k\Sigma _\alpha \frac{1}{{4\pi D_\alpha ^2}}f_\alpha ^k\sigma _i\nu _i^k\varepsilon _i$$

where *N*_D_ describes the number of target protons for the IBD reaction in the detector, $D_\alpha ^2$ is the averaged square of the distance from some reactor volume element *α* to the detector, $f_\alpha ^k$ is the fission rate of isotope *k* in volume element *α*, *σ*_*i*_ is the bin-averaged IBD cross section, *ε*_*i*_ is the detector efficiency for bin *i*, and $\nu _i^k$ is the antineutrino yield per fission of isotope *k* into bin *i*. Antineutrino yields are available for the major heavy metal isotopes using the fission products from a 400 keV fission-inducing neutron; the calculated yield varies very little with incident neutron energy (thermal, 400 keV, 14.1 MeV) compared to the difference between isotopes^20^. The detector event rates are integrated over time to produce the expected count in each bin for each detector.

**Fig. 2:**
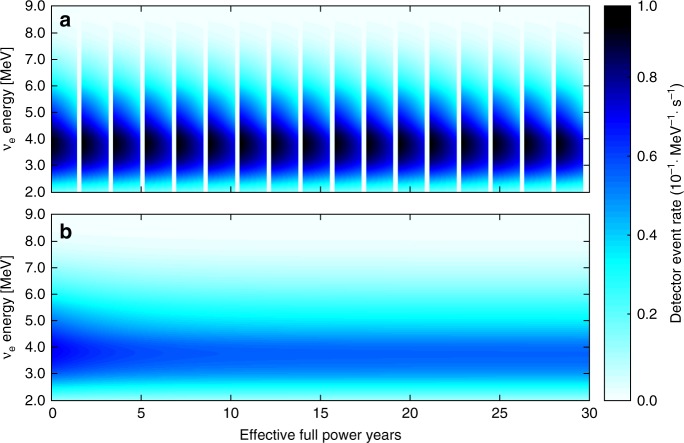
Evolution of antineutrino spectrum and antineutrino detector response as a function of reactor operational time. The emission rates of antineutrino particles at different energies vary with operating lifetime as reactors shift from burning uranium to plutonium. **a** The PWR signal consists of a repeated 18-month operating cycle with a three month refueling interval, while (**b**) the UCFR operates continuously (excluding maintenance interruptions). The UCFR antineutrino signature reaches an equilibrium after ~10 years when plutonium fission dominates and initial enriched uranium reserves are depleted

Under standard operation of both the PWR and UCFR, the transition from primarily fissioning ^235^U to ^239^Pu can be tracked, in line with reactor observation experiments^12^. The higher thermal power rating of the PWR produces a more intense antineutrino field than the UCFR and features a smooth and steady evolution of the signal with the fuel composition over the course of the burnup cycle. The primary difference in the fuel cycles arises from the PWR receiving periodic refueling, in contrast to the continual production of fissile fuel in the fertile column of the UCFR. Once the initial ^235^U inventory is depleted from the UCFR starter fuel, the nominal detector signal enters an approximately steady-state regime in which burn zone propagation, rather than isotopic evolution of the fuel, drives changes in magnitude. During this section—the majority of the UCFR burn cycle—there is negligible change in the nominally detected antineutrino spectrum, and changes in magnitude are much slower and more subtle than during the ^235^U-^239^Pu transition. The evolution of the resulting antineutrino signal from both types of reactors is highlighted in Fig. 2.

The uncertainties affecting the comparison calculation between the antineutrino signal from reference and perturbed reactor states arise from the following elements of the calculation: reactor physics uncertainty *σ*_rp_, antineutrino yield uncertainty *σ*_yields_, detector parameter uncertainty *σ*_det_, reactor operating power uncertainty *σ*_power_ used for diversion masking, and the fitting errors for the burnup-dependent evolution of the calculated nominal reference and perturbed detector event rates *σ*_fit,ref_ and *σ*_fit,div_, as detailed in Supplementary Note 3. These are combined in quadrature to produce the parameter *σ*_norm_ in the *χ*^2^ goodness-of-fit calculation (Eq. (2)). Each uncertainty element may have multiple components, and for the reactor physics uncertainty these components show significant correlations and anti-correlations. Additional information on each of these factors is provided in the Appendix.

The detector background is estimated by scaling the measurement-validated Monte Carlo simulations by the PROSPECT team^19^ up to RETINA-sized detectors. The relevant background in the IBD-based antineutrino detectors of the kind used in RETINA is able to be categorized and quantified:

1. Reactor-correlated background was able to be nearly completely shielded via a multi-layer lead, polyethylene, and boroated polyethylene structure by the PROSPECT team at a standoff of ~7 m from the HFIR reactor used as an antineutrino source. At a standoff of over three times greater distance, this was deemed to contribute negligibly to RETINA background, especially considering that built-in shielding tends to be more consistent and thorough at power reactors than at small research reactors^19^.
2. Uncorrelated gamma coincidences from radioactive decay of nuclei in the surrounding walls, bedrock, etc., which was the primary background for earlier antineutrino detectors, is able to be largely mitigated via detector segmentation and utilizing the outer layer of segments as active shielding. Pulse-shape discrimination (PSD) is able to further improve rejection of uncorrelated gammas.
3. Cosmogenic fast neutrons originating via muon decay can thermalize and be captured in the detector volume in a coincidence that mimics IBD. Pulse-shape discrimination, timing windows based on characteristic delay of the coincident signal, and active rejection via a secondary muon veto detector immediately above the primary detector volume can filter out most cosmogenic background, but it still comprises the vast majority of the IBD-like background. This background is dependent on the altitude of the reactor facility and must be re-calculated for each site. We use the background reported at the HFIR facility (259 m above sea level).

The metric used for calculating detection probability is based on a *χ*^2^ goodness-of-fit test of the perturbed detector signal to the reference signal (Eq. (2)); the departure from nominal average reactor power is considered as a free parameter, *x*. The final term in the summation accounts for the increase in detection probability for prolonged average operation at power above or below declared levels.

2 $$\chi ^2 = \left( {\mathop {\sum}\limits_{\mathrm{b}} {\frac{{\left( {n_{\mathrm{b}} - \left( {1 + x} \right)n_{\mathrm{b}}{\!\!\! \prime} } \right)^2}}{{n_{\mathrm{b}}}}} } \right) + \left( {\frac{x}{{\sigma _{{\mathrm{norm}}}}}} \right)^2$$

The calculated *χ*^2^ represents the expected difference in reference and perturbed detector signal; stochastic processes cause real differences to vary about *T*_0_ = *χ*^2^ as $N\left( {T_0,2\sqrt {T_0} } \right)$. Assuming a null hypothesis of no diverted material and an acceptable false positive rate of *α* = 5%, the detection probability is:

3 $$p\left( {{\mathrm{detection}}} \right) = 1 - \Phi \left( {\frac{{T_{{\mathrm{crit}}}^\alpha - T_0}}{{2\sqrt {T_0} }}} \right),$$

where Φ(*x*) is the cumulative distribution function of the standard normal distribution and $T_{{\mathrm{crit}}}^\alpha$ is the lower bound of ${\int}_T^\infty {\chi ^2} \left( k \right)dk = \alpha$.

The calculation is repeated for the expected detector responses at one, three, and six months post-diversion in order to assess identification probabilities for irradiated material use at the minimum, maximum, and extended conversion timelines, respectively.

### Detecting diversions via antineutrinos

Previous work on antineutrino monitoring of nuclear reactor has relied on simplified point-like core models^21^. Although reactors are generally treated as a point source of antineutrinos due to comparatively large reactor-detector standoffs relative to reactor dimensions, the reactor itself cannot be accurately simulated in low-dimension calculations because of the dynamic response of the neutron field to perturbations in local compositions. The analysis here is based on whole-core, 3-D, neutron transport simulation which accounts for spatial effects of assembly diversions. The methodology employed has been previously demonstrated in sensitivity studies of reactor antineutrino safeguards uncertainties^22^. Diverted assemblies are explicitly modified in the simulation to provide added accuracy to the analysis. We consider diversion of fuel assemblies from inner (-I), middle (-M), and outer (-O) radial positions inside the two reactor cores. This allows to account for difference in neutron importance of different core regions. Irradiated assemblies are replaced with fresh ones and a new antineutrino emission rate is calculated. In addition, proliferators are assumed to be sufficiently sophisticated to attempt to alter the average reactor power covertly in order to minimize the change in detector signal. Care was also taken in different diversion scenarios to ensure that the core remained critical.

### PWR diversions

**Fig. 3:**
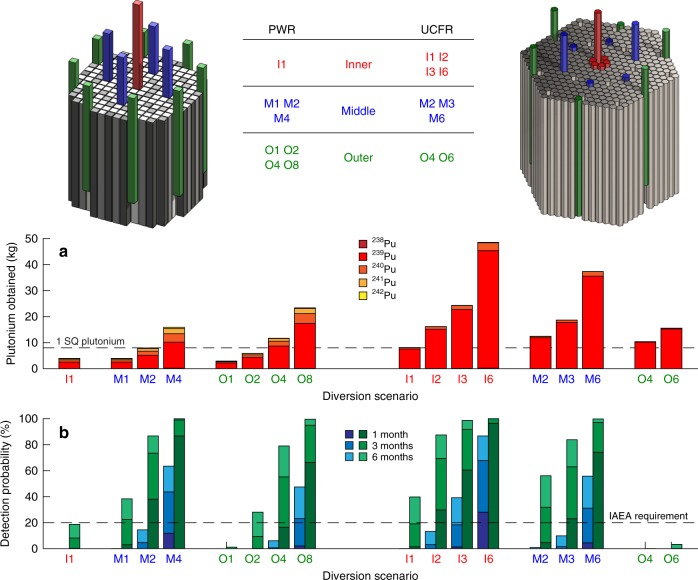
Diversion scenarios for the PWR and UCFR. The scenario identifiers indicate the approximate radial position of the diversion and the number of assemblies removed. Multiple-assembly scenarios assume removal/replacement from symmetric positions in order to maximize separation between perturbed positions, thereby minimizing change in detector signal. The mass of plutonium and its constituent isotopes obtained by a proliferator for each scenario (**a**) depends on whether the assembly has been irradiated for one (outer) or two (inner, middle) cycles prior to removal. The UCFR plutonium composition for all scenarios is weapons-grade, with little contaminant isotopes. The probability of detection via observation by RETINA with count duration of one, three, and six months following the resumption of operations is shown in **b**. The green bars assume no power manipulation by the operator to mask the signal, while blue ones assume power manipulation is performed optimally. Demarcations (dashed lines) indicate the minimum mass required to manufacture a plutonium-based weapon (1 SQ) and the minimum detection probability required by the IAEA for low-probability events (20%)

Regular refueling and assembly shuffling is standard practice in PWR fuel cycles to achieve a relatively flat radial neutron flux shape across the core. The modeled PWR operates under a 3-batch cycle with each assembly residing in the core for three fuel loading. Assemblies are shuffled within the core in a chessboard pattern (highlighted in Fig. 3, with darker assemblies representing higher burnup) to flatten the power density profile. Fuel assemblies are therefore designated either “fresh”,“once-burned”, or “twice-burned”. PWR diversions are assumed to take place during refueling intervals at the end of an operating cycle. An attempt to proliferate during normal operations would require an unplanned shutdown which would be detected by the regulating body. The PWR diversions are of either once-burned (PWR-O) or twice-burned (PWR-I and PWR-M) assemblies. Each type holds a different quantity of plutonium and of varying quality. For instance, once-burnt PWR-O assemblies yield ~3 kg of plutonium (82% fissile), while twice burnt assemblies typically yield just under 4 kg of plutonium at 75% fissile content. These purities are not ideal for weapons production, but can still be attractive to a proliferator. The locations from which assemblies are taken is shown in Fig. 3 along with the reference arrangement of fresh, once-burned, and twice-burned assemblies. In the amounts present at the time of diversion, the PWR-I1, PWR-M1, PWR-O1, (i.e., one assembly from each region) and PWR-O2 (i.e., two assemblies from the outer region) diversions do not provide enough plutonium for a weapon, but a series of such diversions (or parallel diversions from multiple reactor facilities) would eventually aggregate to a weaponizable quantity. PWR-M2 and PWR-O4 therefore represent the minimum material removal for which a single SQ of plutonium is obtained.

### UCFR diversions

The UCFR-1000 core-center position contains a control assembly, so UCFR-I diversions are performed at center-adjacent locations. The central assemblies experience a substantially larger neutron flux than their more peripheral counterparts despite their initially identical fuel compositions. As a result, the inner assemblies contain as much as 1 SQ of plutonium per assembly in just over two years of operation, whereas the outer ones have 1/4 as much. A burnup of 2.17 effective full-power years (EFPY) was chosen for the UCFR-1000 diversions, as this is the earliest a single SQ of plutonium is available via removal of one assembly (any of the six center-adjacent locations). The middle-core and outer-core diversion scenarios were chosen such that a minimum of 1 SQ would be obtained by the actor. Because the fuel is irradiated with a fast neutron spectrum, the plutonium has high purity—the UCFR-I, UCFR-M, and UCFR-O diversions, respectively, yield plutonium with 93, 95, and 98% fissile content.

### Diversion yields and detectability

Analysis of multiple diversion scenarios for both cores (Fig. 3) shows that, in the majority instances, diversions of 1 SQ are detected by a RETINA system with sufficient probability to meet IAEA requirements^17^ for low-probability events, some within one month of resumed reactor operations. While diversions from the PWR core yielding at least one SQ were flagged at rates exceeding the IAEA threshold, sufficient probability was not achieved in three UCFR diversions: UCFR-I1 (one assembly from the inner core regions), as well as UCFR-O4 and UCFR-O6 (four and six assemblies, respectively, from the outer core). The cases with more than 1 SQ of plutonium diverted without detection are summarized in Table 2. The UCFR-I1 diversion can be detected with a 19.1% probability with three months of measurement, approaching the 20% IAEA requirement. The UCFR-O4 and UCFR-O6 diversions, on the other hand, are substantially harder to detect. This is due to the peaked power distribution in the UCFR, resulting in a low neutron importance at the core periphery. The phenomenon is less pronounced in the more mature PWR design, resulting in less spatial variation in fission rates, and by extension, diversion detection probabilities.

**Table 2:** Diversion scenarios and probability of detection

Scenario	*p*(detection) at 1/3 month(s)	Note
ine PWR-O4	16.7%/54.7%	Exceeds 20% at 3 months
UCFR-I1	1.5%/19.1%	Exceeds 20% at 3 months + 4 days
UCFR-M2	4.7%/31.8%	Exceeds 20% at 3 months
UCFR-O4	0.0%/0.0%	Low neutron importance
UCFR-O6	0.0%/0.2%	Low neutron importance

Specifically, detection probabilities under 20% one month after post-diversion measurements, with more than 1 SQ of material diverted, are reported. The most challenging detections are for the outer UCFR scenarios, due to relatively low neutron importance of assemblies in those regions of the core

A skilled reactor operator may attempt to mask the diversion by manipulating the total reactor power (highlighted by the lower, blue-colored values in the diversion bar-plot of Fig. 3). If successful, diversions of more than one SQ may pass undetected. However, such manipulations are very difficult to achieve in practice, and an operator would risk over-correction, which can increase the likelihood of detection relative to the un-masked case. Overshooting the *σ*_power_ range runs the risk of also being detected by traditional safguards monitoring of power level. Furthermore, the operators would have no assurance over the success of their effort. The “flying blind” nature of such manipulation, combined with the potential to inadvertently reduce the chances of maintaining concealment should deter these attempts. Alternatively, while a proliferator may attempt to obtain one SQ in aggregate over the course of several diversions, this is considered an unlikely route due to the risk associated with circumventing traditional safeguards measures on multiple occasions. Consequently, RETINA systems are envisioned to complement current safeguards measures, rather than replace them entirely.

The overall results demonstrate the potential for RETINA systems to safeguard reactor facilities. These detectors can substantially reduce the risk of a weaponizable quantity of material being diverted from current and future reactors. Since the replacement assemblies in all scenarios were the original low-enriched fuel, detection probabilities are expected to increase if the proliferator uses natural uranium instead (a path more likely to avoid IAEA accounting). With regards to the undetected UCFR diversions, the findings can help inform future design decisions of these conceptual cores. To counter the spatial dependence on detection probability, there is a strong imperative for further radial power flattening across assemblies; doing so via design optimization will also bring important safety and burnup advantages as well.

Going forward, the two main areas for further technical improvement in antineutrino-based safeguards are background reduction and narrowing uncertainty on the antineutrino emissions from fission products (the largest component of uncertainty on the signal). As new technology is developed and operational experience gained, background rejection is expected to improve. In parallel, basic nuclear measurements of the kind pursued by SoLid^23^ and PROSPECT^19^ will refine the estimates of the antineutrino flux produced by the aggregate fission products of each fuel species. Additional calibration of measurements using known-state reactors can also be used to enhance precision. All of these improvements can be expected to reduce diversion detection times and increase the probability of detecting the five exception in Table 2.

## Discussion

A wide range of cases can be envisaged with different reactors or diversion schemes. It is considered beyond the scope of the paper to quantify diversion probabilities in each instance, but some discussion on notable cases is provided in this section. In a typical PWR, an alternate diversion scheme could involve the replacement of burnt fuels with natural-uranium bearing ones. This could allow the proliferator to avoid scrutiny by manufacturing fuel from its own uranium deposits, since all fresh fuels supplies are strictly surveilled. Analysis shows that these instances should in fact increase the risk of detection by a RETINA system due to an even larger disturbance in the antineutrino signature. This scenario also runs the risk of not providing sufficient criticality margin at startups or result in an early shutdown of the reactor. Both of which could be detected by traditional safeguarding means. Reducing the risk of detection would require the replacement of an assembly with another plutonium-bearing one, e.g., from the spent fuel pond. This is unlikely since a proliferator would run the risk of detection via traditional safeguards twice (once by displacing the spent fuel, and another form displacing an in-core assembly). A state actor able to divert fuel from the spent fuel ponds is much more likely to use these assemblies for a weapons-program rather than rely on those inside the reactor which are monitored by a RETINA system.

Another potential challenge for a RETINA system arises if a nuclear plant does not operate as baseload. Load-following is increasingly likely as more interment renewable energy sources are added to the grid. In these instances, the reactor power will vary with time, with a correspondingly varying antineutrino source term. Because of the low-statistics regime of a RETINA system, signal detection averaged over days, weeks, or sometimes months is the relevant input to safeguards calculation. When load following is declared, the anticipated antineutrino signature can be adequately adjusted. However, since the overall power would be decreased (load-following typically seldom involves overpower conditions due to safety constraints), the detection time would proportionally increase as a function of $\frac{1}{{\sqrt n }}$ in light of the reduced signal. As a result, if the total reactor energy generated over a period of time is halved, the total number of antineutrino detections is halved, and the detection time needed to draw a conclusion on a possible diversion is doubled. The safeguards regime would have to provide added scrutiny on the reactor during these instances. If on the other hand, load-follow is not declared, a departure from full nominal power can be detected within the order of a few hours via the total antineutrino flux similarly to what was demonstrated in SONGS^12,14^, but at an even faster rate in light of increases in detector efficiency.

One other notable case to consider is online refueling. Many current reactors offer this capabilities (e.g., CANDU, RBMK, AGR). Their assemblies can be removed and replaced individually while reactor power is maintained. The online refueling itself is not a challenge for RETINA at a fundamental level. The system would still follow a similar approach to detect diversions. The main potential issue arises from the fact that these systems make several one-assembly diversions a more attractive route from a logistical standpoint. An SQ of plutonium could be slowly obtained by incrementally diverting one assembly at a time, many months apart. This would result in a much subtler variations in antineutrino signal and would be more challenging to detect with current RETINA technology. Traditional safeguards would need to be relied upon more heavily in these reactors. This issue is exacerbated in the case of the pebble-bed reactor concept. In these advance reactors, small fuel pebbles are continuously cycled in and out of the core. Individual pebbles have low neutronic importance relative to the larger core and their diversion is therefore very difficult to detect. It should be noted however, that monitoring a online-refueling reactor with a RETINA system force a prolifertor to conduct each diversion under large increments of several months or even years to avoid detection. This would substantially increase the breakout time to a weapon.

Many factors come into play when a state decides to pursue nuclear weapons. These dynamics are likely to be significantly altered if antineutrino detectors are universally deployed. First, the continuous presence of “eyes on the ground” will be an important psychological barrier deterring states from proliferating. Second, shortening the detection timeline will provide added opportunity for the international community to intervene before a state can develop a full-fledged weapon from the diverted material. Nuclear proliferation supply-side theorists argue that states are more likely to proliferate if they have the means to do so^24^. The presence of a reactor providing a ready source of fissile material reduces the perceived barriers and costs on the path to proliferation, thus in theory, emboldening decision-makers. In a similar way, the presence of safeguards or antineutrino detectors increases barriers and risks on any attempt to proliferate material. There are no recorded diversion attempts with inspectors present, but instances are believed to have occurred in between inspections (e.g., Iraq 1980s)^25^. The presence of an “always-on” system, while not foolproof, would substantially increase the perceived risk of getting caught attempting to proliferate. As shown in Fig. 3, in very few instances can a potential proliferator confidently extract on SQ of plutonium and avoid detection. Subsequently, RETINA-like systems are expected to deter states from acquiring nuclear weapons, strengthening the nonproliferation regime. Were a diversion to occur, it could be detected on a much shorter timeline than with traditional safeguards. This provides additional opportunity for the international community to intervene by applying diplomatic, economic, and military pressure to dissuade a given state from weaponizing the diverted material.

The deployment path for these near-field antineutrino monitoring systems remains unclear at present. Wadsworth^26^ has explored this issue as it relates to the Islamic Republic of Iran, concluding that certain types of antineutrino detectors should be favored over others, and recommended further investment in R&D efforts. As the author points out, suspect countries are likely to resist the implementation of the RETINA-type systems. Arguments against their deployment might range form “national soveriegnty” concerns to “unnecessary economic burdens”—if the inspectorate is forced to finance the added safeguards. This first issue could be alleviated by some countries setting out the example and voluntarily deploying these detectors on their nuclear fleet. The second issue would be addressed by requiring such a facility would be financed by the IAEA via support from the international community. As an initial step, the next generation of reactors can be designed to include antineutrino detectors with the Nuclear Supplier Group enforcing this added feature. Retrofitting old reactors will prove more difficult as it requires additional economic incentive and goodwill from states. Attempting to produce a universally binding agreement for NPT-signatories is likely to be a difficult path to follow. A more promising route would be to walk along the lines of the voluntary IAEA Additional Protocols, which were gradually enforced worldwide. From 1997 to 2015, the agreement has entered in force in 126 countries^27^. Leveraging this framework, we argue for a phased enforcement of the requirement, based on reactor type and construction time. The first phase of such a protocol would focus initially on new reactor concepts that are most at risk of proliferation (such as the UCFR). In the second phase, any newly planned reactor will need to contain such a system, regardless of type. Then in a last stage, all research and civilian reactors would be retrofitted with monitoring technology. Deploying these detectors to older systems can prove more burdensome, but not impossible, as was demonstrated in the SONGS project^9,12,15,28^. The technical aspect of detectors must also be addressed with standards put in place early on. Detector technology has been improving at a steady rate for the past several years and is expected to keep doing so even if an agreement takes hold. An IAEA certification procedure would need to be implemented, ensuring that as new capabilities are developed, they can be tested and integrated into future deployments.

Beyond the realm of traditional safeguards, RETINA-type systems have also been proposed for disarmament negotiations and plutonium disposition. A recent study has argued for their deployment in Russian fast reactors tasked with eliminating plutonium stockpiles^29^. Studies have also investigated how they can be used to monitor MOX burnup in a PWR to verify plutonium incineration^30^. While the Plutonium Management and Disposition Agreement (PMDA) has recently stalled, antineutrino-monitoring can provide an exciting way of rebuilding trust between states in assuring procedures are being followed. The monitoring systems can also come into play for more ambition international treaties such as the Fissile Material Cutoff Treaty, which is still under negotiation at the UN Conference on Disarmament^31^. The agreement would place a cap on the amount of fissile material produced by nuclear weapons state and would need non-intrusive verification tools for military production reactors. RETINA systems, which collect no sensitive optical or acoustic information, would be ideal for such a task.

The transition of the international nuclear fuel cycle to a hub-spoke model simultaneously reduces the risk of enrichment-based and reprocessing-based proliferation vectors, but the temptation for actors to divert material from the reactor itself remains or may even increase in the case of advanced long-lived designs. Because the RETINA monitoring system relies directly on the antineutrinos emitted from fission fragments, it is protected against spoofing of the relevant physical signature. RETINA-type antineutrino detectors can promptly detect reactor shutdowns, alerting inspectors if an off-cycle interruption occurs. When tasked with detecting compositional changes in the reactor core caused by diversion of material from current systems (PWR), and anticipated future systems (UCFR), the monitors can meet the detection threshold set by the IAEA in the majority of instances when more than one SQ of material is diverted. Notable exceptions were observed for assemblies diverted from the UCFR outer region, and for cases when the plant operator is able to optimally manipulate reactor power output to mask the signal. Further improvements in performance can be expected with future development in background noise rejection, directionality capabilities, detector fudicial volume, reactor-detector standoff, and reduction of uncertainties on detector signal (in particular, the antineutrino yield per fission of fuel isotopes). Despite their limitations, RETINA systems are expected to be an effective deterrent tool against attempted diversion, and therefore warrant further consideration by the IAEA and other agencies concerned with the spread of nuclear weapons.

## Methods

### MCNP models

The Monte Carlo neutron transport code, MCNP6^32^, was used for the modeling of the PWR. The code is well-suited to simulate the performance of thermal cores and considered among the most well-established in the field of nuclear engineering. A core design based on a typical Westinghouse-type PWR was selected for the analysis. Depletion calculations were performed with the newly coupled CINDER90 code^32^. In order to reduce computation costs, the model takes advantage of PWR core symmetry and simulates 1/8th of the core volume. Reflective boundary conditions were applied on either side of the model “slice”. The simulations were all performed for one full operation cycle of 18 months. In order to ensure good statistical accuracy, a total of 100,000 particles were run over 200 cycles for each burn step; resulting in acceptable eigenvalue standard deviations of the order of 20 pcm.

Diversion simulations were performed by modifying the original assembly arrangement and replacing once or twice burnt assemblies in specified locations with fresh ones. The fission rates of the base case was extracted and compared to that of each of the modified diversion simulations. This was used as the basis to calculate the antineutrino response deviance in each diversion scenario highlighted in Fig. 3. Taking advantage of the core symmetry, a total of three diversion simulation were generated in total. Linear interpolation was used for the outer and middle core results in order to deduce the fission rate of other scenarios. The outer core simulation consisted of an eight-assembly diversion while the middle core one consisted of four. Interpolating between each model and the base case was used to obtain the fission rates of PWR-M1, -M2, -O1, -O2, and -O4. For each diversion simulation, the core eigenvalue was ensured to be above critical throughout depletion.

### REBUS models

REBUS is a suite of codes developed at Argonne National Laboratory for fast reactor fuel cycle evaluation. It consists of different modules with specific functionalities. MC^2^-3 generates cross-sections that are weighted by TWODANT, a 2-D S-N code. These cross-sections are then used by the DIF3D-VARIANT, a 3-D P_*N*_ flux solver, to compute the flux in each assembly region. The values are then used by REBUS to calculate fission rates and deplete the isotopic compositions of the fuel. The fission rate evolution over time is then used for the antineutrino detector analysis.

Due to the evolution of the active zone inside of the UCFR-1000, cross-section updating is necessary. An automated script was developed to halt the simulation, re-generate the cross-section file, and then resume the depletion. Because the rate of divergence between the UCFR fuel cycle data should be most pronounced during the beginning of the cycle, metrics from the first 15 Effective Full Power Years (EFPY) were used to assess the case for periodic updates to the effective microscopic cross sections. For the reference fuel cycle, cross sections were updated every 1 EFPY from 0 to 15 EFPY, then every 3 EFPY for the remainder of the cycle. The relaxed update schedule past the first quarter of the burnup cycle is allowed since the burn zone is propagating slowly upward and the change in fuel isotopes is slow compared to at BOC.

To adequately model flux variations inside the reactor, a P_3_ flux angular order was selected. A nodal spatial order of 4 was used for the source term, 6 for the flux term within the node and 1 for the leakage term. A whole core modeled contrary (as opposed to the 1/8th MCNP model). Separate, individual diverted reactor models were run to obtain the relative fission yields; no interpolation between diversion scenarios was conducted. Since the UCFR operates continuously and without any refueling interval, the diversion simulations were taken to begin after 2.17 EFPY of operations. This timeline corresponds to the earliest time a proliferator could acquire 1 SQ of plutonium in a single assembly (I1).

## Supplementary information

Supplementary Info

## Data Availability

The data that support the findings of this study are available on request from the corresponding author A.E. The data are not publicly available due to them being generated using export-controlled codes.

## Code availability

MCNP6 and REBUS codes were used to generate the reactor models and to perform fuel cycle analysis. The codes are available from https://rsicc.ornl.gov.
