# Supplementary material for: Employing antineutrino detectors to safeguard future nuclear reactors from diversions
Source: Nat Commun. 2019 Aug 6;10:3527. doi: 10.1038/s41467-019-11434-z (PMC6684554; doi:10.1038/s41467-019-11434-z)
Supplement: Supplementary file 1 — Supplementary Info [file 41467_2019_11434_MOESM1_ESM.pdf]

Supplementary Information

Employing Antineutrino Detectors to Safeguard Future Nuclear Reactors from Diversions

Christopher Stewart, Abdalla Abou-Jaoude, and Anna Erickson*

*erickson@gatech.edu

Supplementary Note 1: IBD Discussion

Nuclear fission reactions inside of a reactor produce a copious electron antineutrino ($\bar{\nu}_e$) flux. They result from the β^- decay of the fission fragments (Supplementary Equation 1). On average, approximately six such decays occur per fission; at ~ 200 MeV released per fission, there are an order of 10^{20} antineutrinos generated per second per GW_{th} of reactor output.

$$ {}^{A}_{Z}X \rightarrow {}_{Z+1}^{A}Y + \beta^- + \bar{\nu}_e \tag{1} $$

The most mature technology for detecting electron antineutrinos relies on the reverse of this reaction, aptly named inverse beta decay (IBD). The reaction is highlighted in Supplementary Equation 2, it has a cross section on the order of 10^{-43} cm^2 and a threshold energy of 1.8 MeV.[1] This reduces the number of detectable antineutrinos produced per fission to approximately 1.92 for ^{235}U and 1.45 for ^{239}Pu.[2]

$$ \bar{\nu}_{\mathrm{e}} + p \rightarrow e^+ + n \tag{2} $$

Since the IBD reaction does not occur for neutrinos, sources of neutrinos are automatically excluded from detectors using this method. This facilitates monitoring of a nuclear reactor, since the latter emits antineutrino almost exclusively, while the proton chain in the sun produces neutrinos. IBD-based detectors therefore automatically exclude solar neutrinos. This is important since neutrinos, like their antineutrino cousins, cannot be adequately attenuated by matter. Significant design and operation experience has already been gained with small IBD-based antineutrino detectors, allowing them to be deployed in the very near term.

The antineutrino detectors in the LLNL studies at SONGS use gadolinium-doped water as the detection medium – water to supply a large, predictable density of protons for the IBD interaction, and gadolinium to quickly capture the produced neutron.[3–5] The measurement of coinciding positron annihilation and neutron capture is classified as an “antineutrino-like event". The positron can be used to calculate the incident antineutrino energy because its energy signature is measurable and reaction kinematics dictate that the positron carries nearly all of the kinetic energy from the IBD event. Cosmic radiation which might produce a positron or result in a neutron capture in the detector is able to be adequately shielded by taking advantage of the overburden provided by the generally below-grade placement of new reactor cores.[6] MeV-range events, which might also mimic reactor antineutrinos, can be largely mitigated through the use of anti-coincidence.[6]

Supplementary Note 2: Reactor Modeling and Analysis

Two reactor types are considered for the study. One is a conventional Westinghouse PWR, and the other a more advanced concept that does not require any additional fuel throughout its lifetime (so-called ‘breed-and-burn’ design). The second reactor was analyzed to showcase the capability of antineutrino detectors to monitor unconventional concepts as well as more standard ones. The advanced reactor is termed the UCFR-1000; it was developed by the Ulsan Institute of Science and Technology (UNIST) in conjunction with Argonne National Laboratory (ANL).[7] It is a sodium-cooled fast reactor, with a U-10Zr fueled core. Low Enriched Uranium (LEU) at the bottom of the core acts as starter region and sits below a 300 cm fertile region containing natural uranium. The burn zone propagates upward at about 5 $cm.year^{-1}$ as plutonium is bred into the fertile zone, consistent with the CANDLE[8] burnup scheme. It is intended to operate on a once-through cycle which lasts 60 years. The major core parameters are taken from the design paper and are summarized in Supplementary Table 1.[7]

The PWR fuel cycle was modeled in MCNP6,[9] a Monte Carlo code used to simulate neutral particle transport, and the UCFR-1000 in REBUS, a deterministic code developed for modeling fast reactors. The tools were used to obtain the fission rate evolution of each type of actinide as a function of burnup.

The fission rates are converted to expected detector count rates and fit to 2nd-order polynomials to allow for arbitrarily-timed diversions and to smoothly vary the progression of the core composition. Diversions are performed by replacing the existing assembly with a fresh one at the relevant location(s). The data for continued operation in these perturbed states is compared to the reference-state data via nominal detector signal comparison with acceptable envelopes defined by the uncertainty on the signal in each energy bin of the detector to determine the probability of detection in each scenario.

Supplementary Table 1. The main UCFR-1000 core parameters.[7] Core design parameters have a direct effect on its fuel evolution, refueling time and subsequently antineutrino flux.

Parameter	Value
Power Rating (MW_{th}/MW_e)	2600/1000
Cycle Length (EFPY)	60
Fuel Form	U-10Zr
Initial HM Loading (t)	201
Number of Fuel Assemblies	378
Assembly Pitch (cm)	16.5
Fuel Pin Diameter (cm)	1.49
Fuel Smeared Density (%)	74.5

Supplementary Note 3: Sources of Uncertainty

The count rate uncertainty on each individual bin arises from Poisson statistics. The global uncertainty (i.e., that which affects all of the binned counts in a similar manner) has primary contributions from approximation of real-world parameters in the reactor model, errors in the reactor isotopic composition as a function of burnup in the depletion calculation, uncertainties in the reactor power measurement, errors in the isotopic fission rates (also a function of burnup), the isotopic fast-fission antineutrino yields, the IBD cross section, and the detector efficiency. These are weighted by the detector spectrum where applicable and combined in quadrature into a lumped uncertainty σ_{norm} as shown in Supplementary Equation 3. Each of the different sources of uncertainty is detailed in Supplementary Table 2.

$$\sigma^2_{\text{norm}} = \sigma^2_{\text{power}} + \sigma^2_{\text{IBD}} + \sigma^2_{\text{yield}} + \sigma^2_{\text{fit,ref}} + \sigma^2_{\text{fit,div}} \tag{3}$$

Supplementary Table 2. Description and value of the main sources of uncertainty in the antineutrino detector analysis..

**Component**	**Magnitude**	**Note**
σ_{power}	0.2% (UCFR)<br>2% (PWR)	Relative error on measurements of the reactor thermal power output[10]
σ_{IBD}	1.4%	Relative error on the IBD cross section, established in the neutrino physics experiment at Bugey[11]
σ_{yield}	~3.2%	Relative error on the antineutrino yields per fission of each isotope, weighted by isotope and antineutrino energy, and summed over all fissioning isotopes and antineutrino energies
$\sigma_{\text{fit,ref}}$	~1.2%	Normalised RMS error on the evolution of the reference antineutrino source, weighted by bin
$\sigma_{\text{fit,div}}$	~1.2%	Normalised RMS error on the evolution of the diverted-case antineutrino source, weighted by bin

Reactor thermal power is traditionally measured via coolant flow meters in the secondary loop. The development of new instrumentation for measuring the coolant flow is able to reduce the uncertainty on reactor power measurements from current levels of 2% to approximately 0.5% in demonstrated applications and even smaller uncertainties (0.2%) are claimed for similar applications of the technology.[10] Because the UCFR-1000 is at least a decade away from production, it is not unreasonable to assume that 0.2%-level precision on the power measurement is achievable.

The antineutrino yield data for fast fission of the heavy metal isotopes is derived from approximate fast-fission fragment yields and theoretical decay paths; these are currently only known to about 10-20%.[12] However, the difference between fission product yields has been shown to be dominated by the species fissioned rather than the energy of the neutron inducing the fission. Combined with the low (1%) uncertainty[2] on thermal-fission yields of the isotopes undergoing fission in reactors and the negligible difference between the estimated thermal and fast antineutrino yields, it is not unreasonable to assume a similar level of precision will be available for fast fissions of the same isotopes and levels of a few percent for the rarer actinides. The IBD cross section is well-known with a relative uncertainty of 1.4%,[13] and detector efficiency can be estimated with a known

antineutrino emitter upon deployment of the detector system prior to its operation. Any error in the detector efficiency would affect both reference and perturbed cores in a systematic manner.

A χ^2 goodness-of-fit statistic, discussed in the Methods Section of the manuscript, is based on the difference between the integrated detector event rates of the reference ($n_\text{b}(t)$) and perturbed ($n_\text{b}^{'}(t)$) states in each energy bin. The equation accounts for potential attempts by the reactor operator to reduce the difference between the reference state and its post-diversion state, by operating at a different power level. The power manipulation is captured in the parameter x. The last term χ^2 goodness-of-fit statistic (Equation 2 in the main manuscript) applies a penalty to account for the fact that continuous operation above or below the supposed nominal power (under the assumption that an independent statement of the operating power is available), would also raise red flags for a deviation that is high relative to the uncertainty on the power measurement. The analysis essentially measures a worst-case scenario, in which the reactor operator and diverting actor have near-perfect information on the reactor state and the required information to best spoof detection via antineutrinos; something that is unlikely to be the case in reality.

The value of χ^2 applies to the mean of the diverted-case minimized bin difference. In reality, the measured value is distributed about $T_0 = \chi^2$. It was shown that the corresponding statistic T is normally distributed:[13]

$$T \sim N\left(T_0, 2\sqrt{T_0}\right) \tag{4}$$

The null hypothesis in this test is that no nuclear material has been lost. In this context, a false positive (Type-I error) might result in falsely concluding that material has been diverted. Conversely, a false negative (Type-II error) happens when a diversion is concluded to not have occurred when, in fact, it has—this is referred to as the non-detection probability.[14] For a specified critical value of T^{α}_{crit} for which the Type-I error rate is α, the rate of Type-II errors (β) and the power of the test are:

$$\beta = \Phi\left(\frac{T^{\alpha}_{\text{crit}} - T_0}{2\sqrt{T_0}}\right) \tag{5}$$

$$\text{Power} = 1 - \beta \tag{6}$$

where $\Phi(x)$ is the cumulative distribution function of the standard normal distribution. Safeguards tests pre-select α = 0.05 or lower in order to keep the frequency of false alarms low.[14]

The acceptable thresholds for the power of safeguards measurements are 0.9 for high-probability events and 0.2 for low-probability events.[14] The lower threshold in the latter case represents the impetus for action on less certain data due to the generally high consequences of an ignored but real low-probability event. However, the normal distribution of the goodness-of-fit χ^2 results in Power = β = 0.5 at T_0 = T^{α}_{crit}. Should the diversion of material from a functioning reactor be classified as a low-probability event, it is likely that a reduced false-positive threshold would be employed to take advantage of data with non-negligible indication that immediate inspection and/or international intervention is required. However, regularly updated integration of detector events—one of the more intuitive ways to condense time-series safeguards data—would then give many false positives, depending on how often the metric is updated. For example, if the cumulative detector event count is updated daily and the safeguards discrimination has a false positive rate of 0.05, we should expect 18.25 false positive events per year according to the binomial distribution with $n = 365$ and $p = 0.05$. In order to avoid frequent expensive action based on false-positive indications from antineutrino safeguards mechanisms alone, their data should be used in conjunction with other safeguards metrics to allow joint probabilities to guide inspector deployments or corrective measures.

References

1. Vyrodov, V. N. *et al.* Precise measurement of the cross-section for the reaction anti-electron-neutrino + p —> e$^+$ + n at the Bourges reactor. *JETP Lett.* **61**, 163–169 (1995). [Pisma Zh. Eksp. Teor. Fiz.61,161(1995)].
2. Huber, P. & Schwetz, T. Precision spectroscopy with reactor antineutrinos. *Physical Review D* **70**, 053011 (2004).
3. Bowden, N. Reactor monitoring and safeguards using antineutrino detectors. In *Journal of Physics: Conference Series*, vol. 136, 022008 (IOP Publishing, 2008).
4. Bernstein, A., Bowden, N., Misner, A. & Palmer, T. Monitoring the thermal power of nuclear reactors with a prototype cubic meter antineutrino detector. *Journal of Applied Physics* **103**, 074905 (2008).
5. Bowden, N. *et al.* Observation of the isotopic evolution of pressurized water reactor fuel using an antineutrino detector. *Journal of Applied Physics* **105**, 064902 (2009).

6. Bernstein, A. *et al.* Nuclear security applications of antineutrino detectors: current capabilities and future prospects. *Science & Global Security* **18**, 127–192 (2010).

7. Tak, T., Lee, D. & Kim, T. Design of ultralong-cycle fast reactor employing breed-and-burn strategy. *Nuclear Technology* **183**, 427–435 (2013).

8. Sekimoto, H., Ryu, K. & Yoshimura, Y. Candle: the new burnup strategy. *Nuclear Science and Engineering* **139**, 306–317 (2001).

9. Goorley, J. T. *et al. MCNP6 User's Manual* (2013). Los Alamos National Laboratory, LA-CP-13-00634.

10. Djurcic, Z. *et al.* Uncertainties in the anti-neutrino production at nuclear reactors. *Journal of Physics G: Nuclear and Particle Physics* **36**, 045002 (2009).

11. Declais, Y. *et al.* Study of reactor antineutrino interaction with proton at bugey nuclear power plant. *Physics Letters B* **338**, 383–389 (1994).

12. Mueller, T. A. *et al.* Improved predictions of reactor antineutrino spectra. *Physical Review C* **83**, 054615 (2011).

13. Blennow, M., Coloma, P., Huber, P. & Schwetz, T. Quantifying the sensitivity of oscillation experiments to the neutrino mass ordering. *Journal of High Energy Physics* **2014**, 1–41 (2014).

14. IAEA. Glossary Edition, International Nuclear Verification (2001). Austria.
